# Supplementary material for: ReSMAP: Web Server for Predicting Residue-Specific Membrane-Association Propensities of Intrinsically Disordered Proteins
Source: Membranes (Basel). 2022 Aug 11;12(8):773. doi: 10.3390/membranes12080773 (PMC9416665; doi:10.3390/membranes12080773)
Supplement: Supplementary file 1 [file membranes-12-00773-s001.zip › membranes-1839967-supplementary.pdf]

Communication

ReSMAP: Web Server for Predicting Residue-Specific Membrane-Association Propensities of Intrinsically Disordered Proteins

Sanbo Qin [1], Alan Hicks [1], Souvik Dey [1], Ramesh Prasad [1] and Huan-Xiang Zhou [1,2,*]

[1] Department of Chemistry, University of Illinois at Chicago, Chicago, IL 60607, USA
[2] Department of Physics, University of Illinois at Chicago, Chicago, IL 60607, USA
* Correspondence: hzhou43@uic.edu

Table S1. Sequences of eight IDRs.

	Sequence
ChiZ	1 MTPVRPPHTP DPLNLRGPLD GPRWRRAEPA QSRRPGRSRP GGAPLRYHRT GVGMSRTGHG SRPV 64
GluN1	854 AVNVWRKNLQ DRKSGRAEPD PKKKATFRAI TSTLASSFKR RRSSKDTSTG GGRGALQNQK DTVLPRRAIE 923<br>924 REEGQLQLCS RHRES 938
GluN2B	863 SISRGIYSCI HGVAIEERQS VMNSPTATMN NTHSNILRLL RTAKNMANLS GVNGSPQSAL DFIRRESSVY 932<br>933 DISEHRRSFT HS 944
N-WASP	116 QVALNFANEE EAKKFRKAVT DLLGRRQRKS EKRRDPPNGP NLPMATVDIK NPEITTNRFY GPQVNNISHT 185<br>186 KEKKKGKAKK KRLTKADIGT PSNFQHIGHV GWDPN 220
WASP	133 EAQAFRALVQ EKIQKRNQRQ SGDRRQLPPP PTPANEERRG GLPPLPLHPG GDQGGPPVGP LSLGLATVDI 202<br>203 QNPDITSSRY RGLPAPGPSP ADKKRSGKKK ISKADIGAPS GFKHVSHVGW DPQ 255
FtsQ	1 MTEHNEDPQI ERVADDAADE EAVTEPLATE SKDEPAEHPE FEGPRRRARR ERAERRAAQA RATAIEQARR 70<br>71 AAKRRARGQI VSEQNPAKPA ARGVVRGLK 99
SepF1	1 MSTLHKVKAY FGMAPMEDYD DEYYDDRAPS RGYARPRFDD DYGRYDGRDY 50
SepF2	66 ADYPP PGYRGGYADE PRFRPREFDR AEMTRPRFGS WLRNSTRGAL AMDPRRMAMM FEDG 124

Numbering is from full-length proteins. The GluN1 and GluN2B IDRs are tethered to the tetrameric transmembrane domain; each tetramer contains two GluN1 chains and two GluN2B chains, and the MD results were averaged over the two chains. Also, to remove the effects of tethering to transmembrane helices, membrane association results for the 16 residues in the GluN1 IDR and the 19 residues in the GluN2B IDR, preceding the sequences listed here, were not used. SepF2 was never involved in training – it was only used for testing

Table S2. Lipid compositions of membranes in the MD simulations

	Lipid composition
ChiZ	154 POPG; 66 POPE
GluN1	200 POPS; 50 PIP_2; 100 POPC; 100 POPE; 50 Cholesterol
GluN2B	200 POPS; 50 PIP_2; 100 POPC; 100 POPE; 50 Cholesterol
N-WASP	160 POPS; 40 PIP_2; 80 POPC; 80 POPE; 40 Cholesterol
WASP	160 POPS; 40 PIP_2; 80 POPC; 80 POPE; 40 Cholesterol
FtsQ	210 POPG; 90 POPC
SepF1	154 POPG; 66 POPC
SepF2	154 POPG; 66 POPC

Lipid composition is for the leaflet facing the IDRs.

Table S3. Amplitude parameters

	Fully disordered	With stably-bound amphipathic helices
q_+	2.43	2.29
q_-	0.26	0.64
q_0	0.59	1.17

Three other parameters model the distance dependence of the contribution of neighboring residues: $a_\pm$ = 0.0982, $b_\pm$ = 0.00305, and a_0 = 0.521.

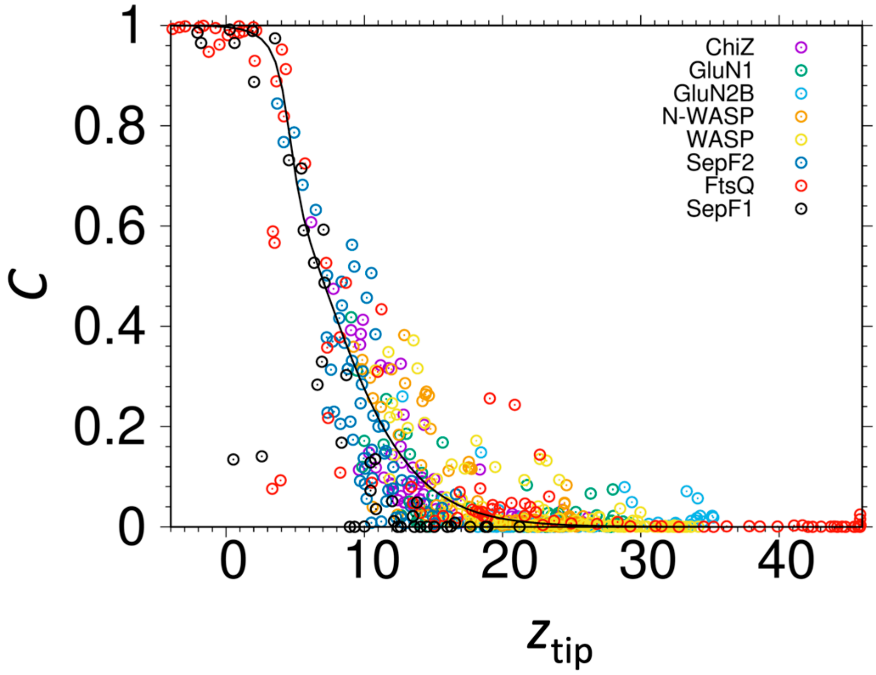

Figure S1. Conversion from z_{tip} to membrane-contact probability. Circles display scatter plots of z_{tip} values and raw contact probabilities of individual residues; the curve displays Equation (1a).

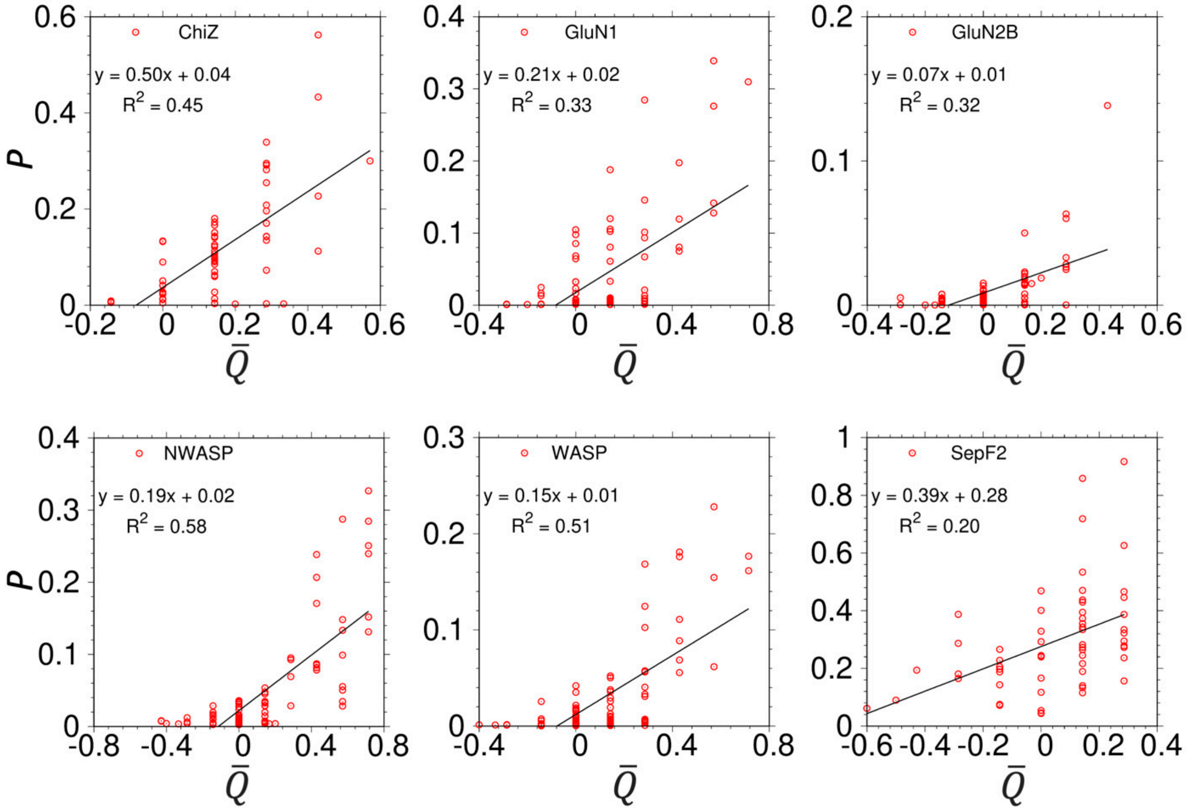

Figure S2. Correlation of MD membrane-contact probabilities (P) with the seven-residue moving average charge ($\bar{Q}$) for six fully disordered IDRs. For each IDR, the line of regression is shown, as are the corresponding equation and the coefficient of determination (R^2). Note that $R^2 = 1 - \mathrm{SSE}/\mathrm{SST}$, where SSE is the sum of squared errors (i.e., difference between observed P and that predicted by the equation of linear regression), and SST is the total sum of squares. The latter squares are over the deviations of P from its mean. For calculating $\bar{Q}$, K, R, and the N-terminus are assigned a charge of +1; D, E, and the C-terminus are assigned a charge of -1; all other amino acids are assigned a charge of 0.

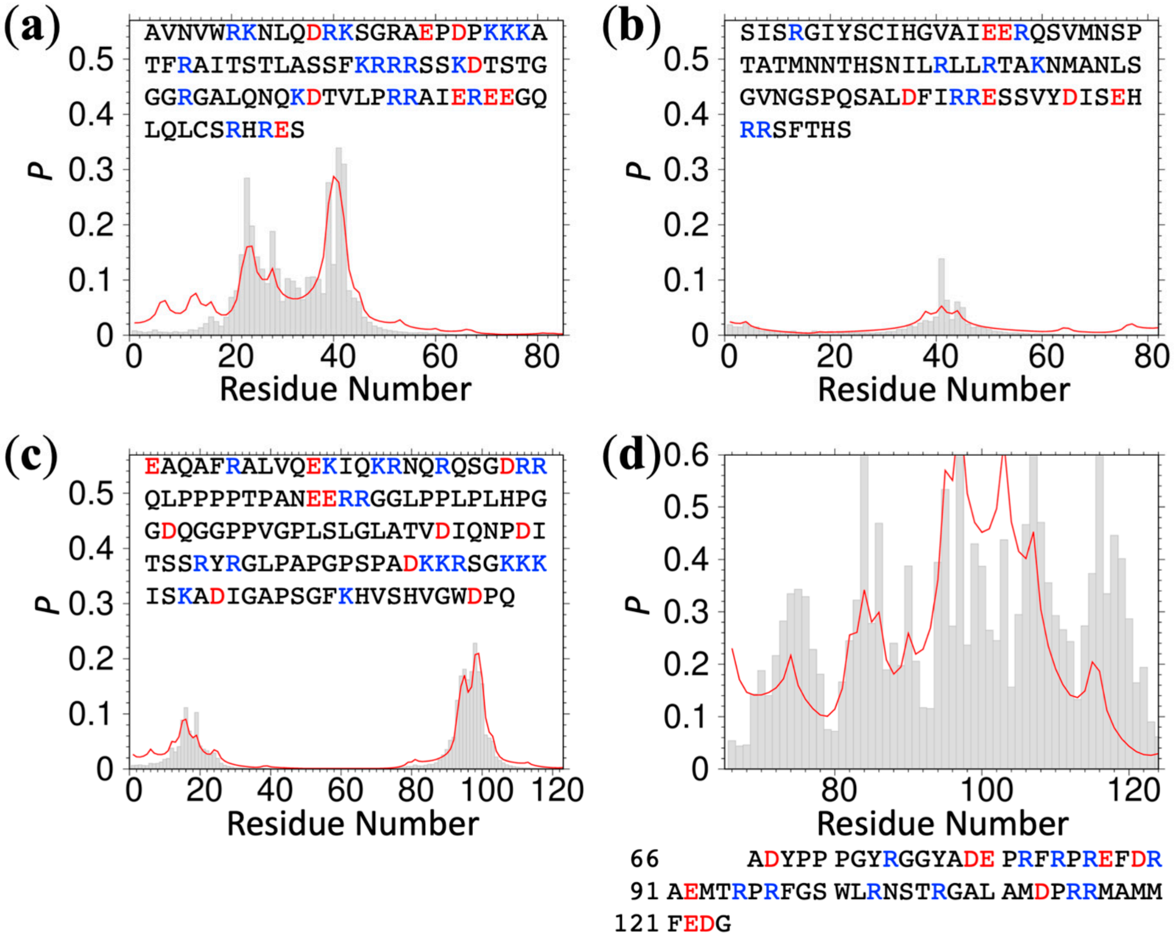

Figure S3. (**a-d**) Comparison of MD membrane-contact probabilities (gray bars) and predicted membrane-association propensities (red curves) for GluN1, GluN2B, WASP, and SepF2, respectively. All these IDRs are fully disordered. The sequence of each IDR is listed, with positively and negatively charged residues colored blue and red, respectively. Note that SepF2 is not in the training set and is thus purely a test IDR.

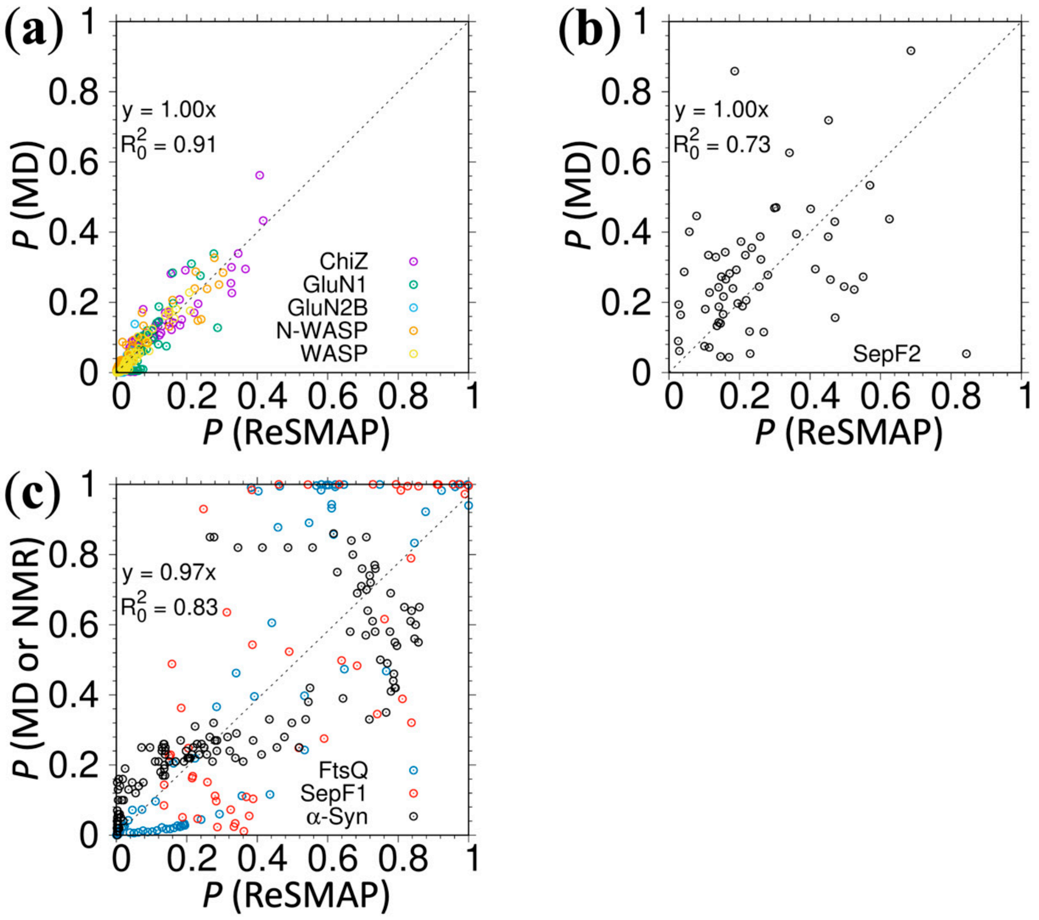

Figure S4. Correlation between MD or NMR membrane-contact probabilities and those predicted by ReSMAP. Regression analysis is carried out with a linear equation without an intercept: $y = ax$, over a single IDR or over data pooled from a set of IDRs. (**a**) The training set of 5 fully disordered IDRs. (**b**) The test IDR SepF2. (**c**) The set of three IDRs with an amphipathic helix. The equation of regression and the coefficient of determination (R_0^2) are displayed as legend. Note that $R_0^2 = 1 - \mathrm{SSE}/SST_0$, where SST_0 is the total sum of squares of observed *P*. SST_0 differs from SST by not subtracting the mean of *P* when calculating squares, and is the appropriate measure for a linear regression equation where the intercept is set to 0. Note that SSE is related to the root-mean-square error (RMSE) via $\mathrm{RMSE} = \sqrt{\mathrm{SSE}/N}$, where *N* is the number of residues, whereas SST_0 measures the mean amplitude of observed *P*. Therefore R_0^2 provides a measure in which the RMSE is compared against the mean amplitude of observed *P*.

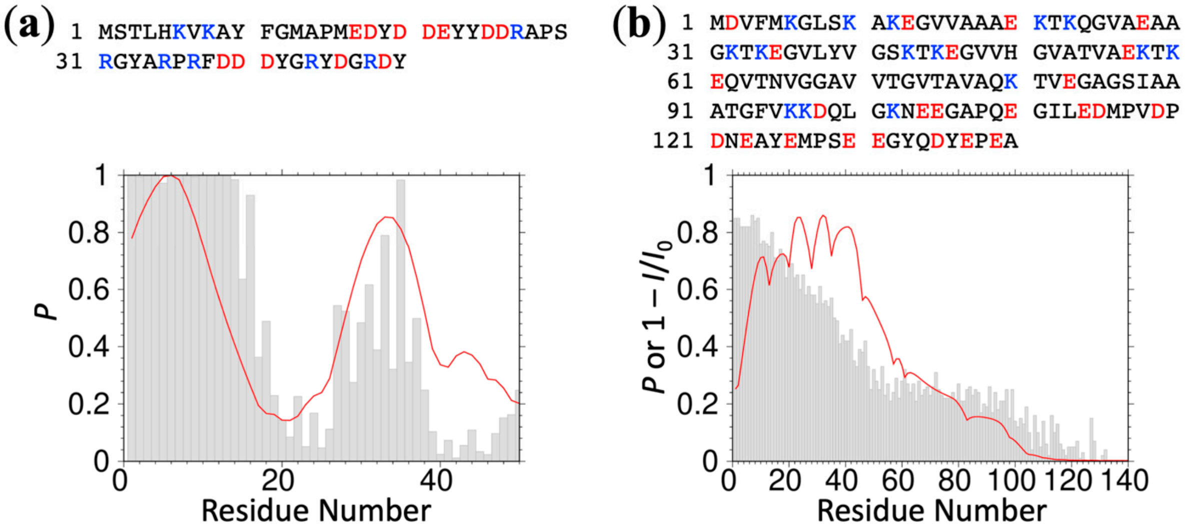

Figure S5. (**a**,**b**) Comparison of MD or NMR membrane-contact probabilities (gray bars) and predicted membrane-association propensities (red curves) for SepF1 and α-synuclein, respectively. These two IDRs have at least one amphipathic helix that stably associates with acidic membranes. The sequence of each IDR is listed, with positively and negatively charged residues colored blue and red, respectively. For α-synuclein, we calculated the membrane-contact probabilities as $1 - I/I_0$, where I_0 and I are NMR peak intensities of the protein in solution and bound to PIP2-vesicles (protein:lipid molar ratio at 1:5), respectively, as reported by Jacob et al. [13].
